# Does Acid Rain Alter the Leaf Anatomy and Photosynthetic Pigments in Urban Trees?

**DOI:** 10.3390/plants9070862

**Published:** 2020-07-08

**Authors:** Verónica M. Rodríguez-Sánchez, Ulises Rosas, Germán Calva-Vásquez, Estela Sandoval-Zapotitla

**Affiliations:** 1 Jardín Botánico, Instituto de Biología, Universidad Nacional Autónoma de México, Ciudad de México 04510, Mexico; vrodriguez@ecologia.unam.mx (V.M.R.-S.); urosas@ib.unam.mx (U.R.); 2 Posgrado en Ciencias Biológicas, Universidad Nacional Autónoma de México, Ciudad de México 04510, Mexico; 3 Laboratorio de Contaminación Atmosférica, Facultad de Estudios Superiores Zaragoza, Universidad Nacional Autónoma de México, Ciudad de México 09230, Mexico; labcont@unam.mx

**Keywords:** leaf anatomy, chlorophyll, *Liquidambar styraciflua*, *Fraxinus uhdei*, cuticle damage, environmental pollution, plant damage, leaf damage, abiotic stress, simulated acid rain

## Abstract

Megapolis such as Mexico City, have atmospheric pollutants that interact with the humidity and solar radiation. The topography of this city promotes air stagnation, generating atmospheric pollutants and episodes of acid rain, a phenomenon well recorded since the end of the 1980s. However, little we know about how urban trees respond to acid rain in the city. Here we present how simulated acid rain causes anatomical and changes in photosynthetic pigments in two of the most abundant urban trees in Mexico City: *Liquidambar styraciflua* L. and *Fraxinus uhdei* (Wenz.) Lingelsh. We first described the leaf anatomy of both species. Then, we used one-year-old trees sprayed with sulfuric acid solutions at pH 2.5 and 3.8, and evaluated visible leaf damage, anatomical alterations, and chlorophyll contents. In both species, the pH 2.5 caused cuticle alterations and areas of total tissue destruction. *L. styraciflua* showed greater sensitivity, but we discuss some of the tolerance mechanisms. Finally, acid rain also reduced the chlorophyll contents. These results contribute toward a catalogue of urban tree species to describe pollution-induced damages, and the identification of tolerant species useful for short- and mid-term detection of environmental crisis, in cities with similar environmental conditions and urban tree composition.

## 1. Introduction

Cities and industrial centers are the source of atmospheric pollutants, which in turn can originate other pollutants through the action of light, temperature, or humidity. An example of this is the acid deposition, which is the accumulation of strong acids and oxides from the atmosphere in the form of rain, snow, gas, or particulates that originate primarily from human activities [1,2]. These substances arise mainly from sulfur sources from the combustion of fossil fuels and the processing of metallic minerals. Another major source is the nitrogen emitted from vehicle use, electricity generation, use of agricultural fertilizers, and cattle [2]. These elements in gaseous form interact with water vapor from the atmosphere, to produce sulfuric and nitric acid [1,2,3,4]. Because of the presence of these acids, acid rain possesses elevated levels of hydrogen ions giving way to pH values below 5.6 [1,2]. Rain usually has a pH of 5.6 because of the dissolved carbon dioxide (CO_2_) which interacts with the water vapor, forming carbonic acid (H_2_CO_3_), making rain slightly acidic [2]. Acid rain is considered a global pollutant, affecting forests, lakes, and farmlands in northern Europe, North America, China, and Mexico [1,2,5]. Mexico City, which is immersed in an endorheic basin, constantly suffers periods of air pollution, such as the “ozone season” during the dry season (November to May), while episodes of acid rain occur in the wet season (June to October). Pollutant gases generated by the socioeconomic activities in Mexico City air are emitted daily. At the same time, there are periodic emission events from the active Popocatépetl volcano (1995–2020), which is 72 km southeast of Mexico City. These volcanic events contribute methane, carbon dioxide, hydrogen sulfide, and water vapor to the atmosphere [6]. It is important to point out that the orographic factors of this area favor the stagnation of pollutants and the formation of acidic compounds that precipitate, of which there have been reports of them since the 1980s [7,8,9,10]. Hence, acid rain events can have serious effects on the health of forests, on the bodies of water that still survive in the region, in addition to the economic impact on the agricultural production areas. Until now, it is known that the forests of the mountain-range Sierra de Ajusco-Chichinautzin and Sierra de las Cruces (which partially surround Mexico City) are under severe stress caused by high levels of ozone, during the dry season and by acid rain in the wet season [6].

In addition, since 1994, the Mexico City government has generated rainfall reports. In these publications, the highest acidity records are always found in the southern part of the city. This pattern is given by the dominant trajectory of winds from north to south. This is an issue because the southern part of the city has the largest conservation surface (87,291 ha), and therefore contains the highest vegetation density in the metropolitan area. Specifically, from 1997 to 2000 Mexico City registered greater acidity from June to August with a pH of 4.2–4.5 [11]. Furthermore, reports since 2006 have shown a gradual increase in acidity, recording pH values below 4 [6,12,13].

According to their chemical composition, in Mexico City sulfates are more prevalent in acid rain than nitrates, unlike patterns that have been observed in the United States, where nitrates are more abundant than sulfates [14]. The studies on acid rain in Mexico City have helped to understand their patterns of occurrence and composition [7,8,9], as well as the rain’s accumulation on the bark of trees in the metropolitan area [10]; however, to date we still lack an understanding on the impact that acid rain has on the leaves, plant anatomy and the physiology of urban trees.

Vegetation is severely affected by acid rain in cities. Acid rain is known to enter the leaf tissue through the cuticle and produce morphological defects in plants [15]. The main visible changes are deformation of the margins and color changes, in addition to the appearance of brown, spotted necrotic lesions [16]. Acid rain generally slows plant growth by generating abnormalities in plant metabolism such as photosynthesis, chlorophyll content, nitrogen metabolism, and production of reactive oxygen species. Inversely, there are also exceptional cases of growth promotion [1,17,18,19,20,21,22,23,24,25,26,27]. Anatomically, acid rain can produce alterations of the cuticle thickness [28,29], cellular deformation and occlusion of stomatal cells, loss of trichomes in the epidermis, cell collapse in the mesophyll, and the formation of scar tissue [30,31].

The impact that acid rain has on the vegetation of cities is one example of the complex phenomena that occur in current urban ecosystems, which require further understanding. Although acid rain events do not directly affect humans, they do affect buildings, water reservoirs, soils, and vegetation. This is even more important as human populations mainly concentrate in large cities, and it has been estimated that by 2050 two-thirds of humanity will live in cities [32].

The impacts of human activities on urban ecosystems can have disastrous consequences for all beings immersed in them. Therefore, measures to characterize the resilience of urban ecosystems must be prioritized, in order to find a balance between human activities and the long-term conservation of natural habitats [33]. For this reason, it is important to better understand the response of plants within urban environments, and their response to pollution episodes. This will allow us to discern between suitable sensitive, indicator, or tolerant species to better design biomonitoring plans in cities. This work aims to describe for the first time the damage that acid rain causes, at lab-simulated pH values like those reported in Mexico City during the wet season, in order to determine leaf morphological and anatomical changes, as well as chlorophyll content, in two frequent tree species.

## 2. Results

**Figure 1:**
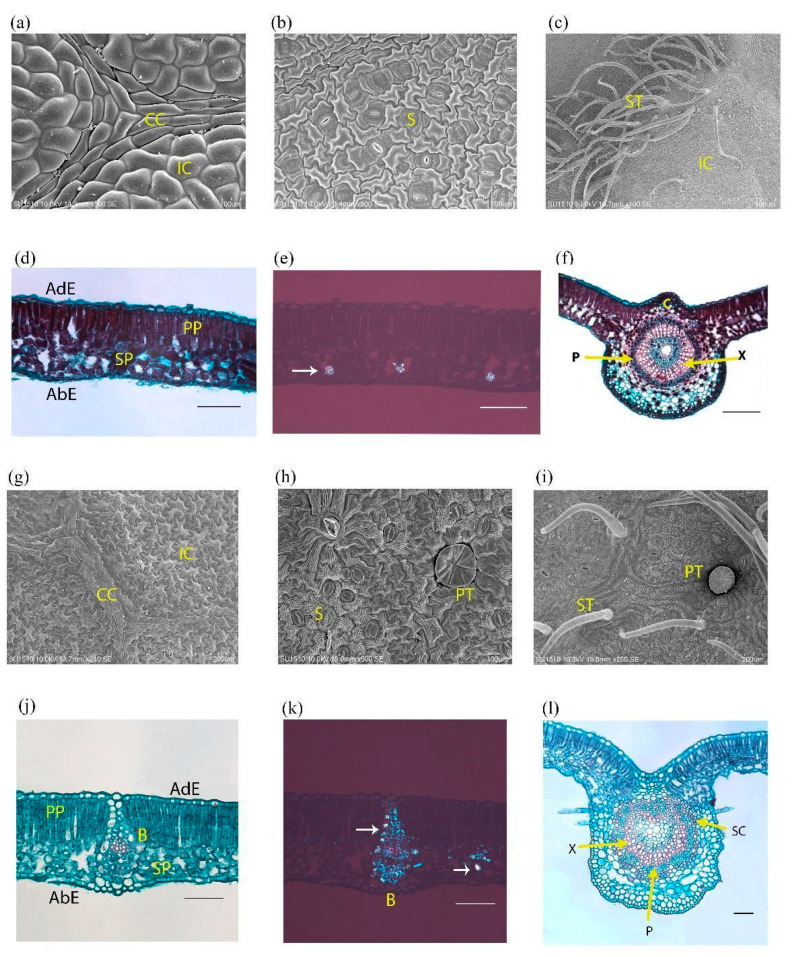
Micromorphology and anatomy of *Liquidambar styraciflua* and *Fraxinus uhdei* leaves. (**a**–**f**) *Liquidambar styraciflua*. (**a**–**c**) Superficial view of the epidermis: (**a**) adaxial surface, (**b**) abaxial surface with simple trichomes, (**c**) paracitic stomata on abaxial surface. (**d**–**f**) Leaf cross section: (**d**) margin, (**e**) druses in the spongy parenchyma, (**f**) middle vein. (**g**–**l**) *Fraxinus uhdei*. (**g**–**i**) Superficial view of the epidermis: (**g**) abaxial surface, (**h**) abaxial surface with simple and peltate trichomes, (**i**) anomocitic stomata on abaxial surface. (**j**-–**l**) Leaf cross section: (**j**) margin, (**k**) stylodes (arrows) in vascular bundle, (**l**) middle vein with simple trichomes, and sclerenchyma (arrow). CC: costal cells; IC: intercostal cells; PP: palisade parenchyma; SP: spongy parenchyma; AdE: adaxial epidermis; AbE: abaxial epidermis; ST: simple trichome; PT: peltate trichome; B: bundle, X: xylem; P: phloem; C: Collenchyma; SC: sclerenchyma; S: stomata (**a**–**c,g,i**) scanning electron microscopy; (**d,f,j,l**) bright field microscopy; (**e**) polarized light; (**k**) phase contrast. Scale represents 100 microns.

In both species, previous descriptions focused on leaf architecture and trichome types, at the genus level. Here we describe the effects of simulated acid rain on the morphology and the anatomy of *L. styraciflua* and *F. uhdei*. We first decided to provide a detailed description of healthy leaves, in these two species (Figure 1).

*Liquidambar styraciflua*. On the leaf surface, epidermal cells can be costal or intercostal (Figure 1a). The costal cells are elongated and rectangular, linear and parallel to the veins. The intercostal cells are irregularly shaped, of various sizes, with sinuous anticline walls on both sides, and smooth outer pericline walls. Its cuticle is also smooth, and epicuticular waxes form aggregates. Leaves are hypostomatic, the stomata are paracitic (Figure 1b). The leaves have unicellular trichomes of constant size and are arranged along the veins (Figure 1c). In a cross-sectional view, the mesophyll is dorsiventral; in both leaf sides, the epidermis has only one layer, but cells are larger on the adaxial surface. There are one to two layers of palisade parenchyma toward the adaxial end. Four to five cell layers of spongy parenchyma are observed toward the abaxial end (Figure 1d). In this tissue, druse crystals are observed in idioblastic cells (Figure 1e). The main vascular bundle is amphicribral (also known as periphloematic). Toward the adaxial end, the main vascular bundle is reinforced by two or three strands of annular collenchyma (Figure 1f). Additionally, abundant cellular contents (presumably tannins) are observed throughout the mesophyll, which have an intense violet coloration after staining (Figure 1f).

*Fraxinus uhdei*. On the leaf surface, epidermal cells are costal or intercostal. The costal cells are elongated and irregular, with linear anticlinal walls. They run parallel to the veins. The intercostal cells are irregularly shaped with sinuous anticlinal walls and are of different sizes (Figure 1g). This condition occurs on both adaxial and abaxial sides of the leaf. They have a striated cuticle, that forms streams of microchannels on the leaf surface. Leaves have glandular peltate trichomes on both sides of the epidermis, with protruding head, constituted by four to eight cells perpendicularly attached to a short unicellular stalk (Figure 1h). Likewise, at the base of the trichome, the cuticular coating is thinner. Leaves have simple trichomes, on the abaxial side near the veins. Leaves are hypostomatic and the stomata are anomocytic (Figure 1h,i). In a cross-sectional view, one can observe that the mesophyll is dorsiventral. On both leaf sides the epidermis has only one layer, but cells are larger in the adaxial surface. There are one to two layers of palisade parenchyma toward the adaxial end. Three to four cell layers of spongy parenchyma are observed toward the abaxial end (Figure 1j). Some of these spongy parenchyma cells have crystals (stylodes), which are also associated with vascular bundles (Figure 1k). Toward the abaxial end of the middle vein, there is parenchyma tissue. It has one to three layers of subepidermal annular collenchyma on both sides of the leaf. The main vascular bundle has an amphicribral arrangement with a sclerenchyma sheath (Figure 1l).

### 2.1. Simulated Acid Rain Causes Visible Leaf Damage

**Figure 2:**
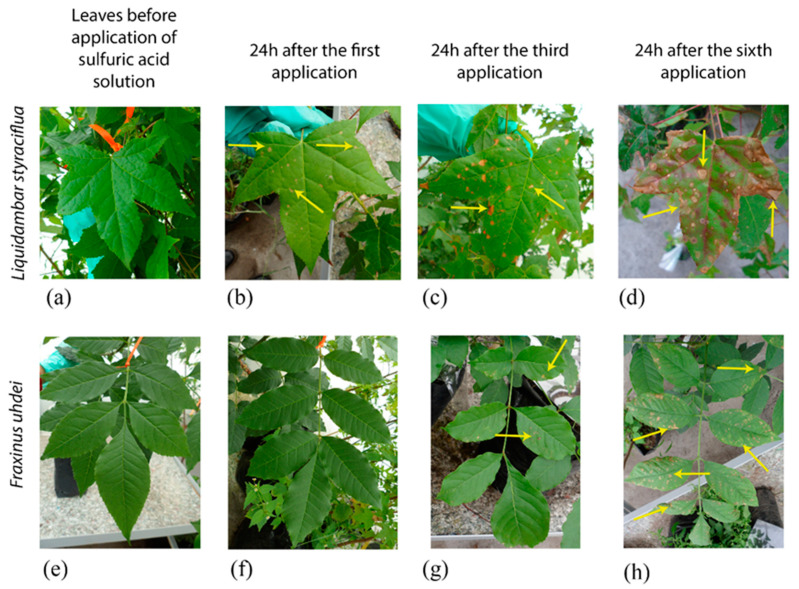
Visible leaf damage of simulated acid rain (pH 2.5). *Liquidambar styracifflua* (**a**–**d**). *Fraxinus uhdei* (**e**–**h**). Arrows indicate some areas of damage caused by the sprayed acidic solution on the margins and the intercostal leaf zones of both species.

In this experiment the morphological effects of acidity on the leaf surface were monitored (Figure 2). Visible leaf damage was only found in the pH 2.5 treatment in both species. Other less severe simulated acid rain treatments (control, pH 5.6 and pH 3.8) did not cause visible morphological leaf damage (Figures S1 and S2). For instance, a treatment with a solution pH 5.6 was performed, but gave no differences as compared to the control group, and therefore it was not further analyzed (data not shown). 24 h after the first spray, *L. styraciflua* displayed brown spots on the intercostal area of the leaves (Figure 2b), whereas *F. uhdei* had a waxier leaf appearance (Figure 2f).

As the experiment progressed, the spots on the leaves of *L. styraciflua* multiplied, spreading all over the leaf also smudging the margins (Figure 2c,d). In contrast, for *F. uhdei* the color of the leaflets started to change after the third application of pH 2.5 acidic solution (Figure 2g). At the end of the experiment, the two species had necrotic spots on the intercostal area and margin spreading over the entire leaf surface (Figure 2d,h).

### 2.2. Simulated Acid Rain Causes Anatomic Leaf Alterations

**Figure 3:**
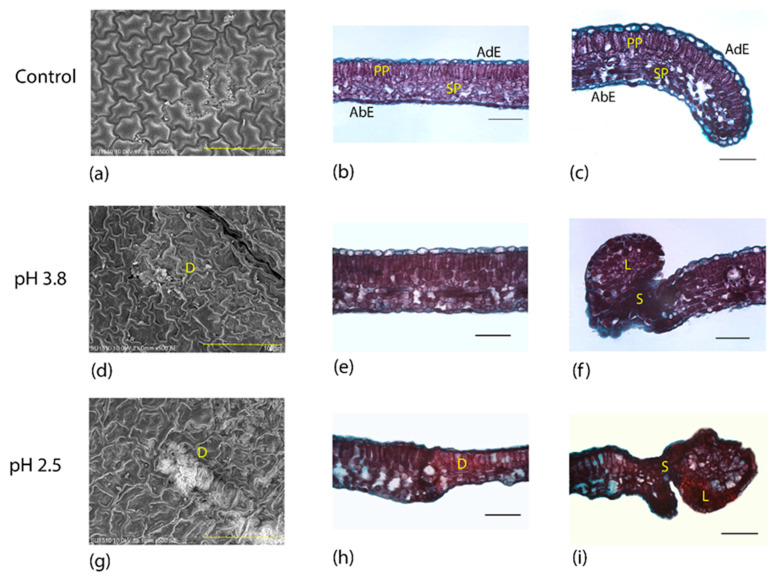
Anatomical changes in leaves of *Liquidambar styraciflua* treated with acid solutions. Control group: (**a**) adaxial view of the epidermis, (**b**) intercostal area, (**c**) margin. Treatment pH 3.8: (**d**) adaxial view of the epidermis, (**e**) intercostal area, (**f**) margin. Treatment pH 2.5: (**g**) adaxial view of the epidermis, (**h**) intercostal area, (**i**) margin. D: damage; S: scar; PP: palisade parenchyma; SP: spongy parenchyma; AdE: adaxial epidermis; AbE: abaxial epidermis; L: lobule; S: scarred tissue (**a,d,g**) scanning electron microscopy; (**b,c,e,f,h,i**) bright field microscopy. Scale represents 100 microns.

Figure 3 shows the control group (a–c) and the anatomical alterations of the *L. styraciflua.* The pH 3.8 treatment caused alterations on the cuticle and collapsed epidermal cells on the adaxial surface, as well as the accumulation of epicuticular waxes in certain intercostal areas (Figure 3d). There was an increase in cellular contents, which enhanced the purple color staining throughout the mesophyll (Figure 3e). Besides, damaged cells became very dark, forming lobules of scarred tissue, which separated these cells from the healthier tissue (Figure S3). The tissue eventually began to acquire an ovoid shape that strangled the lobule finally separating it from the rest of the leaf (Figure 3f). At pH 2.5, the epidermis completely collapsed in intercostal regions, and on the margin of both the adaxial and abaxial surfaces, but aggregates of epicuticular waxes accumulated around the damaged areas (Figure 3g). The staining of the tissues was also intense violet and tissue damage was observed throughout the mesophyll (Figure 3h). In some intercostal and margin areas, the mesophyll partially or totally collapsed. The cells surrounding the sites of greater damage swelled and suberized their walls, creating scars that prevented the spread of lesions. At the margin of the leaf, the tissue formed ovoid structures which later separated from the tissue (Figure 3i).

**Figure 4:**
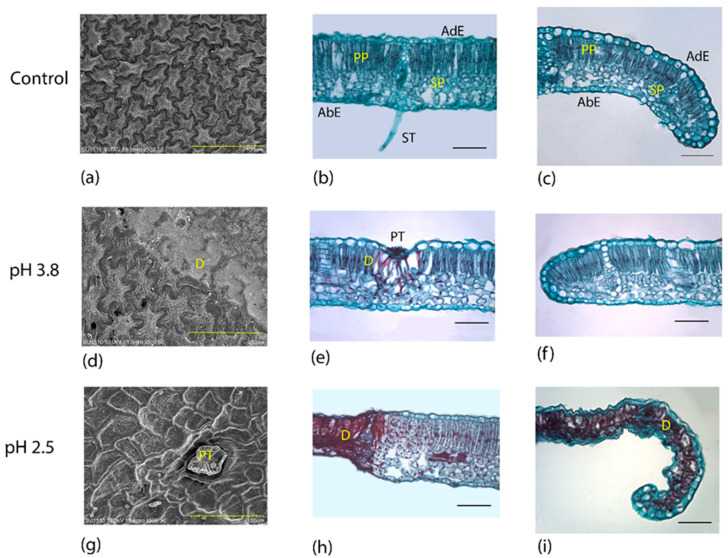
Anatomical changes in the leaves of *Fraxinus uhdei* treated with acid solutions. Control group: (**a**) adaxial view of the epidermis, (**b**) intercostal area, (**c**) margin. Treatment pH 3.8: (**d**) adaxial view of the epidermis, (**e**) intercostal area, (**f**) margin. Treatment pH 2.5: (**g**) adaxial view of the epidermis, (**h**) intercostal area, (**i**) margin. D: damage; PP: palisade parenchyma; SP: spongy parenchyma; AdE: adaxial epidermis; AbE: abaxial epidermis; PT: peltate trichome (**a,d,g**) scanning electron microscopy; (**b,c,e,f,h,i**) bright field microscopy. Scale represents 100 microns.

On the other hand, Figure 4 shows the control group (a–c) for *F. uhdei*. Alterations were only observed on the adaxial surface of the leaflets. In the treatment of pH 3.8, there was an accumulation of epicuticular waxes on the drying marks of the acid droplets, so that only the striated cuticular pattern in certain areas was lost (Figure 4d). Another marking of damage was found at the base of the peltate trichomes on the adaxial surface, due to the cuticular thinning at the base of trichomes (Figure 4e). There were no alterations at the margin of the leaf (Figure 4). For the 2.5 treatment, there were deformations in the structure of the peltate trichomes, and loss of the cuticle, together with the collapse of the epidermal cells in intercostal areas and at the margin of the leaf (Figure 4g). Internally, there was total tissue damage in some areas of the margin, and the mesophyll of intercostal areas completely collapsed. Damage was mainly present in the palisade parenchyma. The appearance of cellular compounds was also observed, expanding from the damaged to the healthy tissue, forming dark spots stained in red, which highlight the damaged cells and tissue (Figure 4h). Lastly, some margins showed total mesophyll collapse (Figure 4i).

### 2.3. Simulated Acid Rain Causes Changes in Chlorophyll a and b Content

**Figure 5:**
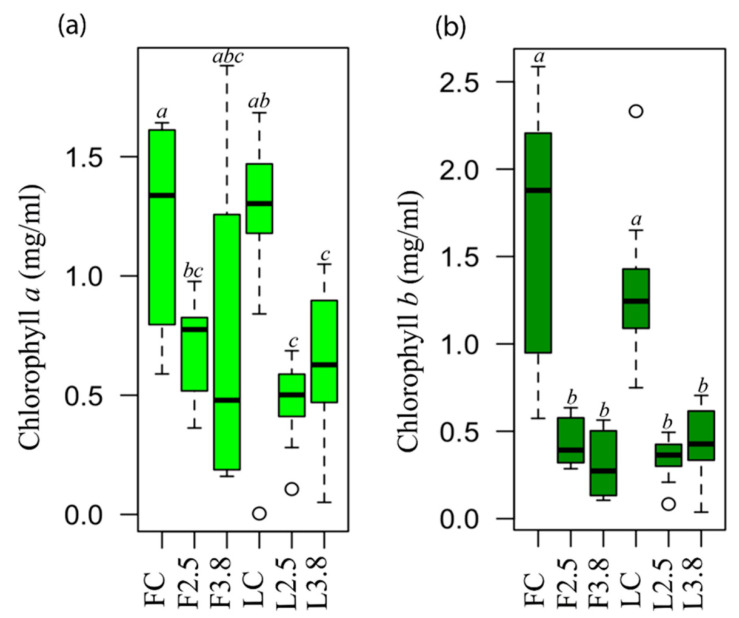
Chlorophyll *a* and *b* concentration leaves treated with acid solutions. (**a**) chlorophyll *a*, (**b**) chlorophyll *b*. *Fraxinus uhdei:* FC (Control), F2.5 (pH 2.5), F3.8 (pH 3.8). *Liquidambar styraciflua*: LC (Control), L2.5 (pH 2.5), L3.8 (pH 3.8). Statistically homogeneous groups (HSD-Tukey-test *p* < 0.05) are indicated with letters. n = 10 per species per treatment.

To evaluate a proxy of the photosynthetic capacity of leaves under simulated acid rain, chlorophyll *a* and *b* content was measured. A Shapiro-Wilk normality test was performed and provided that most levels were normal (Supplementary Table S1), we performed a one-way ANOVA with six levels on each chlorophyll. It was found that in both species, there was a reduction of at least 1.5-fold in chlorophyll *a* (F_(5,54)_ = 6.41; *p* < 0.001) and *b* (F_(5,54)_ = 23.99; *p* < 0.001) content in simulated acid rain treated leaves (Figure 5). The same analysis was also performed for the chlorophyll content per fresh weight, giving similar results (Figure S4). This probably reflects the severe damage observed at pH 2.5, although it is not consistent with the non-visible or anatomical damage at pH 3.8. This indicates that physiological damage might be present, even though morphological or anatomical damage might not be seen.

## 3. Discussion

This research shows for the first time the impact of acid rain levels reported for Mexico City on the anatomy of two abundant tree species. Visible morphological damage was only observed in simulated acid rain at pH 2.5, suggesting that anatomical damage precedes morphological damage. Anatomical damage in both species was observed when simulated acid rain was pH 3.8 and 2.5, but the most severe was on the latter. Acid rain reduced the chlorophyll *a* and *b* content in both species, even when visible damage was not observed at pH 3.8, indicating that physiological damage precedes anatomical damage.

We acknowledge that these results were obtained in greenhouse-controlled conditions, however, the effects of acid rain in real urban environments could be different and remain to be addressed. Despite the frequent acid rain during the rainy season in Mexico City, both species are still part of the urban landscape, probably because they have acid rain tolerance strategies. Based on our results, we propose tolerance mechanisms for each species. In *L. styraciflua* there might be two mechanisms: first, leaves create scars to contain the damage, and second leaves abscise damaged margins which are later shed. In *F. uhdei*, the leaves have a striated cuticle, which possibly allows for less damage at pH 3.8, because the cuticle facilitates the slide down of fluids from the leaf blade. However, this was not the case at the base of peltate trichomes, where the cuticle is thin, non-striated, and damage at pH 3.8 was severe. Overall, our data contributes to imprint our understanding of how acid rain affects urban vegetation, and raises alarm in order to reduce acid rain production in urban centers such as Mexico City. Moreover, it raises concern on better ways to monitor the effect of acid rain on vegetation, as it cannot be easily detected by just looking at visible leaf damages.

Previous studies found that the intensity of leaf damage depends on the concentration of the sprinkled acids [16,18,30,31,34], in which visible damage appears when acidity is below pH 3. The damages we found in the leaves of *L. styraciflua* at pH 2.5, were the necrosis of tissue forming spots in the intercostal area, some distorted margin areas, similar to those found in transgenic *Populus tremula* (Salicaceae) and *Betula pubescens* (Betulaceae), in which their resistance to the herbicide phosphinothricin was tested [35,36]. Another found alteration was the abscission of some leaves; this is consistent with previous reports for this species at pH 3 [37]. Similarly, *Genipa americana* L. (Rubiaceae) sprinkled with acids pH 3, showed the appearance of necrotic spots on the adaxial surface just 24 h after the first application, as well as suberized cells that form scars as a protective barrier to the advancement of cellular necrosis [31], consistent with what we found in *L. styraciflua*.

For *F. uhdei* in the pH 2.5 treatment, the first symptoms of damage began right after the third acid application, similar to *Gallesia integrifolia* (Phytolaccaceae), *Mimosa artemisiana* (Fabaceae), and *Spondias dulcis* (Anacardiaceae), which were sprayed during ten consecutive days with an acid solution pH 3, and started to show damages on the third round of application [38]. In our experiment, the damage symptoms showed yellow chlorotic mottling, which in some cases ended up in the formation of necrotized tissue in intercostal spaces of the leaflets. This was similar to *Ligustrum lucidum* (Oleaceae), which belongs to the same family of *F. uhdei*, in which a treatment of pH 2.5 caused leaf damage shown as yellow or brown spots in the intercostal area, as well as the appearance of marginal necrosis [39,40].

Regarding the anatomical damage in both species in the pH 2.5 treatment, there was cellular collapse of the margin, and total or partial destruction of the mesophyll in intercostal areas, without affecting the vascular bundles. Similar anatomical damage from simulated acid rain has been reported in *G. americana* [31], *G. integrifolia*, *M. artemisiana* and *S. dulcis* [38], *Eugenia uniflora* (Myrtaceae), and *Clusia hilariana* (Clusiaceae) [41] *Paubrasilia echinata* (Fabaceae) and *Libidibia ferrea* var. *Leiostachya* (Fabaceae) [42], subjected to pH 3 simulated acid rain treatments.

It is important to highlight that all the leaf areas affected in the treatment at pH 2.5 on both species, were intensely stained with safranin. This dye is cationic with an affinity for acidic substances. The intense safranin staining could be caused by an increase in cellular contents that act as defense mechanisms against stress caused by acid rain [30]. These cellular contents are likely phenolic compounds, which are a mechanism of response to the increase in cellular acidity or some other stress factor [38,43,44]. Moreover, these compounds are accumulated mainly in the central vacuole of the cells, and can be released into the cytoplasm in case of a tissue alteration caused by some environmental factors [45,46].

A key factor for the generation of lesions was the orientation and characteristics of the leaves [16,30,47,48,49,50]. This is because the emergence and expansion of lesions from acid rain can be related to the contact time of acid drops with the leaf surface, which is also affected by the morphological characteristics of the leaves, such as the type of margin, type of venation, size of veins, or distribution of veins. It is important to note that in both species, the damage was greater in the intercostal areas. In this region there are no cells with lignified cell walls, such as in the coastal region (vascular tissue), and therefore cells in this region are more susceptible to damage. Damage is generated on the margin because it is the region most exposed to acidity, because of the sliding and accumulation of rain droplets.

Despite the absence of visible damage in the pH 3.8 treatment, when making micromorphological observations of the leaves, alterations were found on the cuticle of both species, indicating that micromorphological alterations could be used for prognosis of lesions, as has been previously proposed by others for simulated acid rain or monitoring of urban areas [38]. Both species showed alterations in the cuticles because of the generation of wax clusters where acid droplets were accumulated. When leaves are exposed to the acid, the acid is able to oxidize and hydrolyze the wax esters, releasing some long-chain fatty acids from the cuticular waxy matrix, therefore forming aggregates on the leaf surface [51]. We observed this phenomenon mostly in pH 2.5, wherein the cuticle was affected at the sites of contact and accumulation of acid drops, generating the formation of amorphous wax aggregates, cuticle scaling, the erosion of epicuticular waxes, and the loss of turgor in the cells of the epidermis. These alterations have also been found in *G. americana* [31], *G. integrifolia*, *M. artemisiana* and *S. dulcis* [38], *E. uniflora* and *C. hilariana* [41], *P. echinata* and *L. ferrea* var. *Leiostachya* [42] and *Joannesia princeps* (Euphorbiaceae) [52].

Furthermore, changes on the leaf surface, and particularly the cuticle, could generate a hydrophilic condition that increases the water permeability, making the leaf more sensitive to water loss, or to the entry of acidic substances into the tissues [34,53,54,55]. For instance, we report damage at the base of trichomes in *F. uhdei* when treated with pH 2.5; this could be due to laxer cuticles at these sites as compared to the rest of the leaf surface. This thinning in the cuticle has been previously reported [16,56], which explains the existence of cuticle pores where the cuticle is thinner, through which certain substances penetrate more easily into the leaf. Additionally, it is possible that as water drops evaporated, acid concentration increased, eventually becoming an even stronger acid that causes lesions in the epidermal tissue as previously reported [57].

The cuticle is an important factor for the protection of internal tissues, as well as for photosynthetic pigments [29,51,58]. Moreover, acid rain at pH 4.5 decreased the net photosynthetic rate and Hill-reaction rate in soybean leaves [59]. In this cited case, the acidity levels were high. The acidic substances came into contact with the chloroplasts of the palisade chlorenchyma, the photosynthetic pigments underwent changes in their structure, which in turn led to a decrease in photosynthetic pigments concentration and reduced efficiency to capture electrons [59]. Therefore, the leaf chlorophyll content is an important indicator of direct foliage damage and is strongly related to plant productivity, which can be substantially reduced by acid rain [60]. This is what we observed at pH 2.5, where chlorophyll *a* and *b* concentrations decreased (Figure 5). This has been previously explained as the decrease in chlorophyll concentration may reflect their degradation, because acid rain generates an imbalance in H^+^ ions in leaf cells [61]. In addition, the increase in H^+^ ions could displace Mg^2+^ from the chlorophyll molecule, turning into pheophytin [62]. Finally, the decrease in chlorophyll *a* and *b* depends upon the pH of the simulated acid rain and the exposure time [63]. However, the biochemical alterations that occur during acid rain are far from being fully understood.

It is important to highlight that the scientific literature is mostly focused on species of agricultural interest. Nevertheless, given the expansion of cities and the constant need to improve the environmental quality for its inhabitants (humans, plants and animals), research should be carried out that evaluate the morphological, anatomical, histochemical, and physiological aspects of resilience in these urban species. For instance, trees not only provide environmental services, such as reduction of city temperatures, reduction of wind speed, noise reduction, or harbor other species, but also can serve as indicators of adverse environmental conditions that can pose a threat to human health. The importance of understanding urban environments has been recently recognized within the emerging field of urban ecology [64]. In this sense, this work contributes toward identifying potential urban species, as bioindicators, or resistant species not only to acid rain but to other atmospheric pollutants present in cities, and thus generate planting and replacement programs in urban parks to improve air quality.

## 4. Materials and Methods

### 4.1. Species Selection

The species were selected based on an inventory of the green areas of Mexico City [65]. We decided to use 80 cm-tall, 1-year-old trees from *Liquidambar styraciflua* and *Fraxinus uhdei*, obtained from Viveros Coyoacán, Mexico City (CDMX). These plant age and size are usually used for reforestation and naturation projects in the city. Then, they were transferred to an experimental greenhouse of the Botanical Garden, Institute of Biology, UNAM, CDMX. On this site, the trees were acclimatized for three months at an average temperature of 28.8 °C, and an average relative humidity of 88. Simultaneously, commercial fertilizer (Vigoro, Mexico City, Mexico) was applied so that the trees were in optimal nutritional conditions. After acclimatization, trees were distributed on three tables of 1 m^2^; each table corresponded to one treatment and contained five trees per species (n = 30).

### 4.2. Simulated Acid Rain

We used sulfuric acid solutions with a pH of 3.8, a value reported for Mexico City. We also simulated a scenario with increased acidity (pH of 2.5), a mild acidity (pH 5.6; data not shown), and a control treatment (distilled water pH 7) for baseline comparisons. Before beginning the experiment, the substrate of each tree was covered with an acetate film to avoid the effects acidity on the roots.

The spraying of the solutions was done six times over two weeks of July, on alternate days to allow for the trees to recover. The total sprinkled volume was 30 mm (l × m^−2^), equivalent to 5 mm× day^−1^, which resembles a two-months period of the rainy season in Mexico City [14]. Although the rainy season in Mexico City goes from May to October, August and September are the months with acid rain records with pH below 5.6, and therefore the sprinkled solutions attempted to resemble this two-month exposure of plants to acid rain. Simultaneously, we recorded visible leaf damage.

### 4.3. Sample Collection

To observe the damage caused by simulated acid rain on the surface and inside laminar tissues, seven samples of leaves or leaflets (in *F. uhdei*) were taken at random from four individuals per species and treatment. Then, a subdivision of these leaves was done with the following criteria: the right-central portion of the leaf was taken, which was then divided into three 1 cm^2^ portions: the middle vein, the intercostal area, and the margin zone. For quantification of chlorophyll *a* and *b*, a complete leaf or leaflets was taken from each species and treatment, on each of the 10 replicates, and fresh weight was recorded prior chlorophyll quantification.

### 4.4. Cuticle Patterns

To observe the laminar surface and cuticular patterns, leaf samples were fixed in FAA (formaldehyde—acetic acid—ethanol) solution for 24 h, then rinsed in distilled water and dehydrated in an ethanol series (30%, 50%, 60%, 70%, 80%, 95%, and 100%). Samples were dried to critical-point with CO_2_ for one hour with a dryer Quorum K850 (Quorum Technologies Ltd., Laughton, UK). Then samples were mounted on aluminum holders with carbon conductive tape, and finally covered with gold for two minutes at 20 μÅ with a Quorum Q150R (Quorum Technologies Ltd., Laughton, UK). Observations were made on a Hitachi SU1510 scanning electron microscope (Hitachi High Technologies America, Inc., Schaumburg, USA) at Laboratorio Nacional de Biodiversidad (LaNaBio, Mexico City, Mexico) at Instituto de Biología, UNAM.

### 4.5. Anatomical Damage

To observe the damage inside the tissues, samples were processed according to previous protocols [66], in which leaves or leaflets were fixed in a FAA solution for 24 h, then rinsed with distilled water. They were then dehydrated in a series of tertiary butyl alcohol-ethanol-water mixes (Table S2). Subsequently, they were infiltrated and embedded in histological paraffin at 58 °C; paraffin blocks were obtained, and samples were oriented to obtain cross sections.

The 15-μm thick histological sections were cut in an American Optical 820 rotation microtome (US), stained with safranine-fast green, and finally mounted on synthetic resin. Observations were made using an Axioskope photomicroscope (Carl Zeiss, Oberkochen, Germany), and photographs were taken from the middle vein, intercostal area, and margin from each species and treatment, using a video camera Exwave HAD (Sony, Tokyo, Japan). Finally, the photographs were edited in the GIMP 2.10.6 software, to remove some stains in the preparation-ns.

### 4.6. Quantification of Chlorophyll a and b

The concentration of chlorophyll *a* and *b* was quantified using complete leaves, following a previously published method [67]. Samples were macerated immediately after collection in a solvent solution of acetone-hexane (4:6) to perform chlorophyll extraction. Next, the absorbance of each sample was measured at 645 nm for chlorophyll *a,* and 663 nm for chlorophyll *b* using a Milton Roy Spectronic 20D spectrophotometer (Milton Roy, Ivyland, USA). Subsequently, we used equations [67] to obtain the concentration in mg * 100 mL^−1^ of chlorophyll *a* and *b*. Finally, the data were tested for normality using the Shapiro-Wilk test, followed by a one-way ANOVA with post-hoc HSD-Tukey test in R [68], to show statistical differences (*p* < 0.05) in chlorophyll concentrations for each species and treatments. The equivalent statistical analysis was done on chlorophyll content expressed per unit of fresh weight (chlorophyll mg × g^−1^).

## 5. Conclusions

Acid rain alterations on the leaf anatomy depend on the acid concentration and the leaf exposure time. Only visible damage occurred at pH 2.5 treatment for both species; however, anatomical and biochemical damage was detected at pH 3.8. Damage was observed as necrotic and chlorotic spots from yellow to brown in the intercostal spaces, at the bases of trichomes, and the margin of the leaves.

The cuticle of both species underwent alterations when in contact with acid drops, including cuticle scaling and the formation of epicuticular wax aggregates. Tissues were also damaged, including slight cellular alterations, formation of scars and total cell collapse, except in the vascular bundles. Finally, the concentration of chlorophylls *a* and *b* decreased as acidity treatments increased in both species.

*L. styraciflua* was more susceptible to simulated acid rain than *F. uhdei*. Nonetheless, *L. styraciflua* has tolerance mechanisms such as the generation of scars and the abscission of damaged tissue.

## Supplementary Materials

The following are available online at https://www.mdpi.com/2223-7747/9/7/862/s1, Figure S1. Visible leaf damage of simulated acid rain in the control group. *Liquidambar styracifflua* (a–d). *Fraxinus uhdei* (e–h). Figure S2. Visible leaf damage of simulated acid rain in pH 3.8 treatment. *Liquidambar styracifflua* (a–d). *Fraxinus uhdei* (e–h) Figure S3. Formation of lobules of scarred tissue (a–c) and margin separation (d-f) in *Liquidambar styraciflua* at the pH 3.8 treatment. Figure S4. Chlorophyll *a* and *b* content in leaves per unit of fresh weight. Table S1. Shapiro-Wilk test of chlorophyll *a* and *b* data shown in Figure 5. Table S2. Series of mixtures for tissue dehydration [55].

## References

[1] Abbasi T., Poornima P., Kannadasan T. (2013). Acid rain: Past, present, and future. Int. J. Environ. Eng..

[2] Clair T., Burns D., Rosas-Pérez I., Blais J., Percy K., Hidy G.M., Brook J.R., Demerjian K.L., Molina L.T., Pennell W.T., Scheffe R.D. (2011). Ecosystems. Technical Challenges of Multipollutant Air Quality Management.

[3] Jalali M., Naderi E. (2012). The impact of acid rain on phosphorus leaching from a sandy loam calcareous soil of western Iran. Environ. Earth Sci..

[4] Li Z.X., Yang W.J., Ahammed G.J., Shen C., Yan P., Li X., Han W.Y. (2016). Developmental changes in carbon and nitrogen metabolism affect tea quality in different leaf position. Plant Physiol. Biochem..

[5] Ma Y., Wang B., Zhang R., Gao Y., Zhang X., Li Y., Zuo Z. (2019). Initial simulated acid rain impacts reactive oxygen species metabolism and photosynthetic abilities in *Cinnamonum camphora* undergoing high temperature. Ind. Crop. Prod..

[6] Calva G. (2012). Dendroquímica En Estudios Ecológicos Y Ambientales.

[7] Aguilar S., Bravo H., Saavedra I., Torres R. (1984). Acid precipitation in Mexico City Basin. Proceedings of the Technical Conference: Urban Climatology and It Applications with Special Regard to Tropical Areas.

[8] Báez P.A., Padilla G.H., De González O.G. (1986). Acid Rain Over Mexico City Valley and Surrounding Rural Areas. Geofis. Int..

[9] Peñaranda L.F. (1987). Precipitaciones Ácidas: Metodología Para Su Caracterización Y Estudio De La Ciudad De México.

[10] Alvarado F., García L. (1988). Estudio De La Lluvia Ácida En Corteza De Árbol Como Indicador De Contaminantes En El Volcán El Pelado. D. F.

[11] Secretaría Del Medio Ambiente De La Ciudad De México (2000). Informe Anual De La Calidad Del Aire Y Precipitación Ácida En El Valle De México. http://www.aire.cdmx.gob.mx/descargas/publicaciones/flippingbook/informe_anual_calidad_aire_2000/.

[12] Secretaría Del Medio Ambiente De La Ciudad De México (2012). Calidad Del Aire En La Ciudad De México, Informe 2011. http://www.aire.cdmx.gob.mx/descargas/publicaciones/flippingbook/informe_anual_calidad_aire_2011/#p=1.

[13] Secretaría Del Medio Ambiente De La Ciudad De México (2018). Calidad Del Aire En La Ciudad De México, Informe 2017. Dirección General De Gestión De La Calidad Del Aire, Dirección de Monitoreo Atmosférico. Ciudad De México. http://www.aire.cdmx.gob.mx/descargas/publicaciones/flippingbook/informe_anual_calidad_aire_2017/mobile/#p=1.

[14] Secretaría del Medio Ambiente de la Ciudad de México (2016). Calidad del aire en la Ciudad de México, informe 2015. Dirección General de Gestión de la Calidad del Aire, Dirección de Monitoreo Atmosférico. México, D.F.. http://www.aire.cdmx.gob.mx/descargas/publicaciones/flippingbook/informe-2015-calidad-del-aire-en-la-ciudad-de-mexico/mobile/index.html.

[15] Amthor J. (1984). Does acid rain influence plant growth? Some comments and Observations. Environ. Pol. Ser. A.

[16] Evans L.S., Gmur N.F., Da Costa F. (1977). Leaf surface and histological perturbations of leaves of *Phaseolus vulgaris* and *Helianthus annuus* after exposure to simulated acid rain. Am. J. Bot..

[17] Evans L.S. (1984). Botanical aspects of Acidic precipitation. Bot. Rev..

[18] Birdi K.S., Larsen B.R. (1987). Y Sánchez, R. Effects of simulated acid rain on the surface tension of selected leaves. Colloid Polym. Sci..

[19] Fuzhu Z., Xiaofeng Y., Jinyang Z. (1994). Effects of simulated acid rain on the injury and physiological responses of crops. J. Environ. Sci..

[20] Soukupová J., Albrechtova J., Svobodová H., Opatrná J. (2002). Anatomical and histochemical changes of Norway spruce buds induced by simulated acid rain. Biol. Plant..

[21] Bamidele J.F., Eguagie M.O. (2015). Ecophysiological response of *Capsicum annuum* L. exposed to simulated acid rain. Niger. J. Biotechnol..

[22] Liu M., Yi L., Yu F., Yin X. (2015). Chlorophyll fluorescence characteristics and the growth response of *Elaeocarpus glabripetalus* to simulated acid rain. Photosynthetica.

[23] Sun J., Hu H., Li Y., Wang L., Zhou Q., Huang X. (2016). Effects and mechanism of acid rain on plant chloroplast ATP synthase. Environ. Sci. Pollut. Res..

[24] Fei J., Ma J., Yang J., Liang Y., Ke Y., Yao L., Li Y., Liu D., Min X. (2019). Effect of simulated acid rain on stability of arsenic calcium residue in residue field. Environ. Geochem. Health.

[25] Zheng W., Li R., Yang Q., Zhang W., Huang K., Guan X., Wang S. (2019). Short-term response of soil respiration to simulated acid rain in *Cunninghamia lanceolata* and *Michelia macclurei* plantations. J. Soils Sedim..

[26] Huang J., Wang H., Zhong Y., Huang J., Fu X., Wang L., Teng W. (2019). Growth and physiological response of an endangered tree, *Horsfieldia hainanensis* merr to simulated sulfuric and nitric acid rain in southern China. Plant Physiol. Biochem..

[27] Zhang C., Yi X., Gao X., Wang M., Shao C., Lv Z., Chena J., Liu Z., Shen C. (2020). Physiological and biochemical responses of tea seedlings (*Camellia sinensis*) to simulated acid rain conditions. Ecotoxicol. Environ. Safe.

[28] Cape J.N. (1986). Effects of air pollution on the chemistry of surface waxes of Scots Pine. Water Air Soil Pollut..

[29] Hull H.M., Morton H.L., Wharre J.R. (1975). Environmental influences on cuticle development and resultant foliar penetration. Bot. Rev..

[30] Da Silva L., Alves A., Da Silva E., Oliva M. (2005). Effects of simulated acid rain on the growth of five Brazilian tree species and anatomy of the most sensitive species (*Joannesia princeps*). Aust. J. Bot..

[31] Sant’Anna-Santos B., Campos da Silva L., Alves A., Aguiar R. (2006). Effects of Simulated Acid Rain on Leaf Anatomy and Micromorphology of *Genipa americana* L. (Rubiaceae). Braz. Arch. Biol. Technol..

[32] Martin-Moreau M., Ménascé D. Urban Resilience: Introducing This Issue and Summarizing the Discussions, Field Actions Science Reports [Online], Special Issue 18 2018, Online since 15 December 2018, Connection on 08 May 2019. https://journals.openedition.org/factsreports/pdf/4629.

[33] Alberti M., Marzluff J.M. (2004). Ecological resilience in urban ecosystems: Linking urban patterns to human and ecological functions. Urban. Ecosyst..

[34] Baker E.A., Hunt G.M. (1986). Erosion of waxes from leaf surfaces by simulated rain. New Phytol..

[35] Lebedev V.G., Faskhiev V.N., Kovalenko N.P., Shestibratov K.A., Miroshnikov A.I. (2016). Testing Transgenic Aspen Plants with bar Gene for Herbicide Resistance under Semi-natural Conditions. Acta Nat..

[36] Lebedev V.G., Krutovsky K.V., Shestibratov K.A. (2019). Effect of Phosphinothricin on Transgenic Downy Birch (*Betula pubescens* Ehrh.) Containing bar or GS1 Genes. Forests.

[37] Neufeld H.S., Jernstedt J.A., Haines B.L. (1985). Direct foliar effects of simulated acid rain. New Phytol..

[38] Sant’Anna-Santos B.F., Campos da Silva L., Alves-Azevedo A., Marcos de Araújo J., Figueiredo-Alves E., Monteiro da Silva E.A., Aguiar R. (2006). Effects of simulated acid rain on the foliar micromorphology and anatomy of tree tropical species. Environ. Exp. Bot..

[39] Houbao F., Chuanrong L. (1999). Effects of simulated acid rain on seedling emergence and growth of five broad-leaved species. J. For. Res..

[40] Fan H.B., Hang Y.H. (2000). Effects of simulated acid rain on germination, foliar damage, chlorophyll contents and seedling growth of five hardwood species growing in China. For. Ecol. Manag..

[41] Da Silva L.C., Oliva M.A., Azevedo A.A., Araújo J., Aguiar M. (2005). Micromorphological and anatomical alterations caused by simulated acid rain in Restinga plants: *Eugenia uniflora* and *Clusia hilariana*. Water Air Soil Pollut..

[42] Andrade G.C., Silva L.C. (2017). Responses of tropical legumes from the Brazilian Atlantic Rainforest to simulated acid rain. Protoplasma.

[43] Zobel A.M., Yunus M., Iqbal M. (1996). Phenolic Compounds Against in Defense Air Pollution. Plant. Response to Air Pollution.

[44] Vermerris W., Nicholson R. (2008). Biosynthesis of Phenolic Compounds. Phenolic Compound Biochemistry.

[45] Zobel A., Nighswander J.E. (1991). Accumulation of phenolic compounds in the necrotic areas of Austrian and red pine needles after spraying with sulphuric acid: A possible bioindicator of air pollution. New Phytol..

[46] Khoddami A., Wilkes M.A., Roberts T.H. (2013). Techniques for analysis of plant phenolic compounds. Molecules.

[47] Abouguendia Z.M., Baschak L.A. (1987). Response of two western Canadian conifers to simulated acidic precipitation. Water Air Soil Pollut..

[48] Adams C.M., Hutchinson A. (1984). Comparison of the Ability of Leaf Surfaces of Three Species to Neutralize Acidic Rain Drops. New Phytol..

[49] Wellburn A. (1988). Air Pollution and Acid Rain: The Biological Impact.

[50] Kerstiens G., Yunus M., Iqbal M. (1996). Barrier Properties of the Cuticle to Water, Solutes and Pest and Pathogen Penetration in Leaves of Plants Grown in Polluted Atmospheres. Plant. Response to Air Pollution.

[51] Medeiros C.D., Falcaõ H.M., Almeida-Cortez J., Santos D., Santos M.G. (2017). Leaf epicuticular wax content changes under different rainfall regimes, and its removal affects the leaf chlorophyll content and gas exchanges of *Aspidosperma pyrifolium* in a seasonally dry tropical forest. S. Afr. J. Bot..

[52] Carvalho-Andrade G., Nalon-Castro L., Campos da Silva L. (2020). Micromorphological alterations induced by simulated acid rain on the leaf surface of *Joannesia princeps* Vell. (Euphorbiaceae). Ecol. Indic..

[53] Fogg G.E. (1948). Adhesion of water to the external surfaces of leaves. Discuss. Faraday Soc..

[54] Percy K.E., Baker E.A. (1987). Effects of simulated acid rain on production, morphology and composition of epicuticular wax and on cuticular membrane development. New Phytol..

[55] Eichert T., Fernández V., Marschner P. (2012). Uptake and release of elements by leaves and other aerial plant parts. Marschner’s Mineral. Nutrition of Higher Plants.

[56] Martin J.T., Juniper B.E. (1970). The Cuticles of Plants.

[57] Evans L.S., Curry T.M. (1979). Differential responses of plant foliage to simulated acid rain. Am. J. Bot..

[58] Koch K., Bhushan B., Ensikat H.J., Barthlott W. (2009). Self-healing of voids in the wax coating on plant surfaces. Philos. Trans. R. Soc. A.

[59] Hu H., Wang L., Liao C., Fan C., Zhou Q., Huang X. (2014). Combined Effects of Lead and Acid Rain on Photosynthesis in Soybean Seedlings. Biol. Trace Elem. Res..

[60] Du E., Dong D., Zeng X., Sun Z., Jiang X., De Vries W. (2017). Direct effect of acid rain on leaf chlorophyll content of terrestrial plants in China. Sci. Total Environ..

[61] Shan Y.F., Feng Z.W., Yang H.X. (1989). Effects of Simulated Acid Rain on Seedlings of *Cyclobalano glauca* Oerst. Acid Rain and Agriculture.

[62] Shan Y. (1998). Effects of simulated acid rain on *Pinus densiflora*: Inhibition of net photosynthesis by the pheophytization of chlorophyll. Water Air Soil Pollut..

[63] Kumari P., Tomar Y.S. (2009). Effect of stimulated acid rain on chlorophyll and ascorbic acid contents of *Mentha piperata* (Pepperiment). Agric. Sci. Digest..

[64] Mathey J., Rößler S., Lehmann I., Bräuer A., Otto-Zimmermann K. (2011). Urban Green Spaces: Potentials and Constraints for Urban Adaptation to Climate Change. Resilient Cities.Cities and Adaptation to Climate Change, Proceedings of the Global Forum 2010.

[65] Chacalo A., Corona V., Esparza N. (2009). Árboles Y Arbustos Para Aiudades.

[66] Sandoval E., Rojas A., Guzmán C., Carmona L., Ponce R., León C., Loyola C., Vallejo M., Medina A. (2005). Técnicas Aplicadas a La Anatomía Vegetal.

[67] Nagata M., Yamashita I. (1992). Simple method for simultaneous determination of chlorophyll and carotenoids in tomato fruit. J. Jpn. Soc. Food Sci..

[68] R Core Team (2019). R: A Language and Environment for Statistical Computing.

