# Supplementary material for: Does Acid Rain Alter the Leaf Anatomy and Photosynthetic Pigments in Urban Trees?
Source: Plants (Basel). 2020 Jul 8;9(7):862. doi: 10.3390/plants9070862 (PMC7411892; doi:10.3390/plants9070862)
Supplement: Supplementary file 1 [file plants-09-00862-s001.zip › supplementary files/Figure S4.docx]

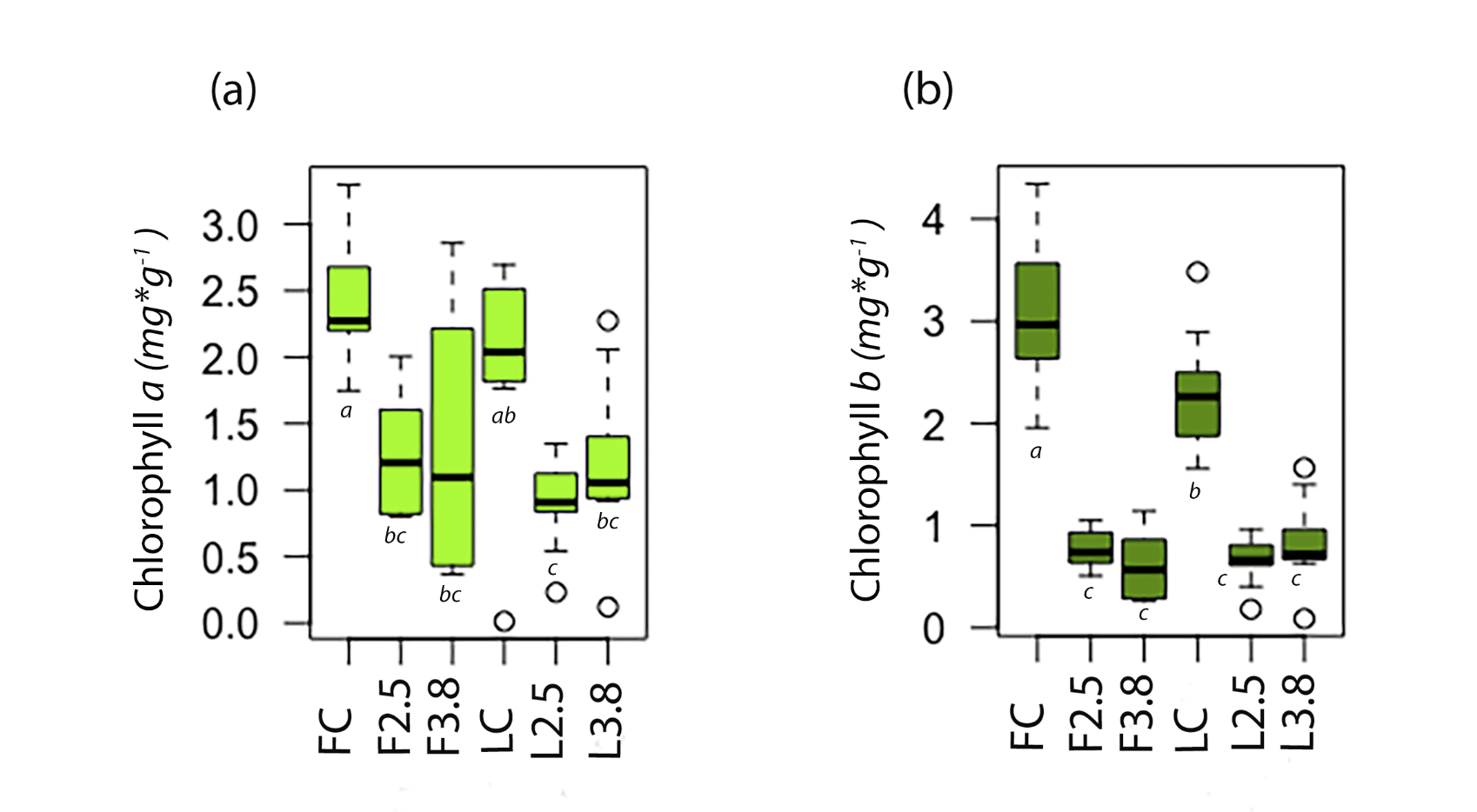


**Figure S4.** Chlorophyll a and b content in leaves per unit of fresh weight. (a) Chlorophyll a, (b) chlorophyll b. Fraxinus uhdei: FC (Control), F2.5 (pH 2.5), F3.8 (pH 3.8). Liquidambar styraciflua: LC (Control), L2.5 (pH 2.5), L3.8 (pH 3.8). One-way ANOVAs showed statistical differences in chlorophyll a (F_(5,54)_=8.063, p<0.001) and chlorophyll b (F_(5,54)_=54.96, p<0.001) content. Statistically homogeneous groups (HSD Tukey-test p<0.05) are indicated with letters. n=10 per species per treatment.
