# Supplementary material for: Does Acid Rain Alter the Leaf Anatomy and Photosynthetic Pigments in Urban Trees?
Source: Plants (Basel). 2020 Jul 8;9(7):862. doi: 10.3390/plants9070862 (PMC7411892; doi:10.3390/plants9070862)
Supplement: Supplementary file 1 [file plants-09-00862-s001.zip › supplementary files/Table S1_Shapiro-Wil test for normality in the chlorophyll a and b datasets..docx]

| Table S1. Shapiro-Wil test for normality in the chlorophyll *a* and *b* datasets. | | | | |
| --- | --- | --- | --- | --- |
|  | Chlorophyll *a* | | Chlorophyll *b* | |
|  | W-statistic | *p*-value | W-statistic | *p*-value |
| FC | 0.87421 | 0.1119 | 0.90575 | 0.2531 |
| F2.5 | 0.90546 | 0.2513 | 0.85703 | 0.07038 |
| F3.8 | 0.86597 | 0.08969 | 0.83668 | 0.04026 |
| LC | 0.79833 | 0.01384 | 0.8914 | 0.1758 |
| L2.5 | 0.92547 | 0.4048 | 0.92008 | 0.3576 |
| L3.8 | 0.9546 | 0.723 | 0.94556 | 0.6164 |
