# Supplementary material for: Does Acid Rain Alter the Leaf Anatomy and Photosynthetic Pigments in Urban Trees?
Source: Plants (Basel). 2020 Jul 8;9(7):862. doi: 10.3390/plants9070862 (PMC7411892; doi:10.3390/plants9070862)
Supplement: Supplementary file 1 [file plants-09-00862-s001.zip › supplementary files/Table S2_Series of mixtures for tissue dehydration [55]..docx]

**Table S2. Series of mixtures for tissue dehydration [55].**

| Tertiary butyl alcohol (ml) | Absolute ethanol  (ml) | Ethanol 96% (ml) | Distilled water (ml) | Final concentration (for 100ml) |
| --- | --- | --- | --- | --- |
| 5 | 0 | 30 | 65 | 35% |
| 10 | 0 | 40 | 50 | 50% |
| 15 | 0 | 45 | 40 | 60% |
| 20 | 0 | 50 | 30 | 70% |
| 35 | 0 | 50 | 15 | 85% |
| 55 | 0 | 45 | 0 | 95% |
| 75 | 25 | 0 | 0 | 100% |
| 100 | 0 | 0 | 0 | Absolute |
