# Supplementary figures and images for: Does Acid Rain Alter the Leaf Anatomy and Photosynthetic Pigments in Urban Trees?
Source: Plants (Basel). 2020 Jul 8;9(7):862. doi: 10.3390/plants9070862 (PMC7411892; doi:10.3390/plants9070862)

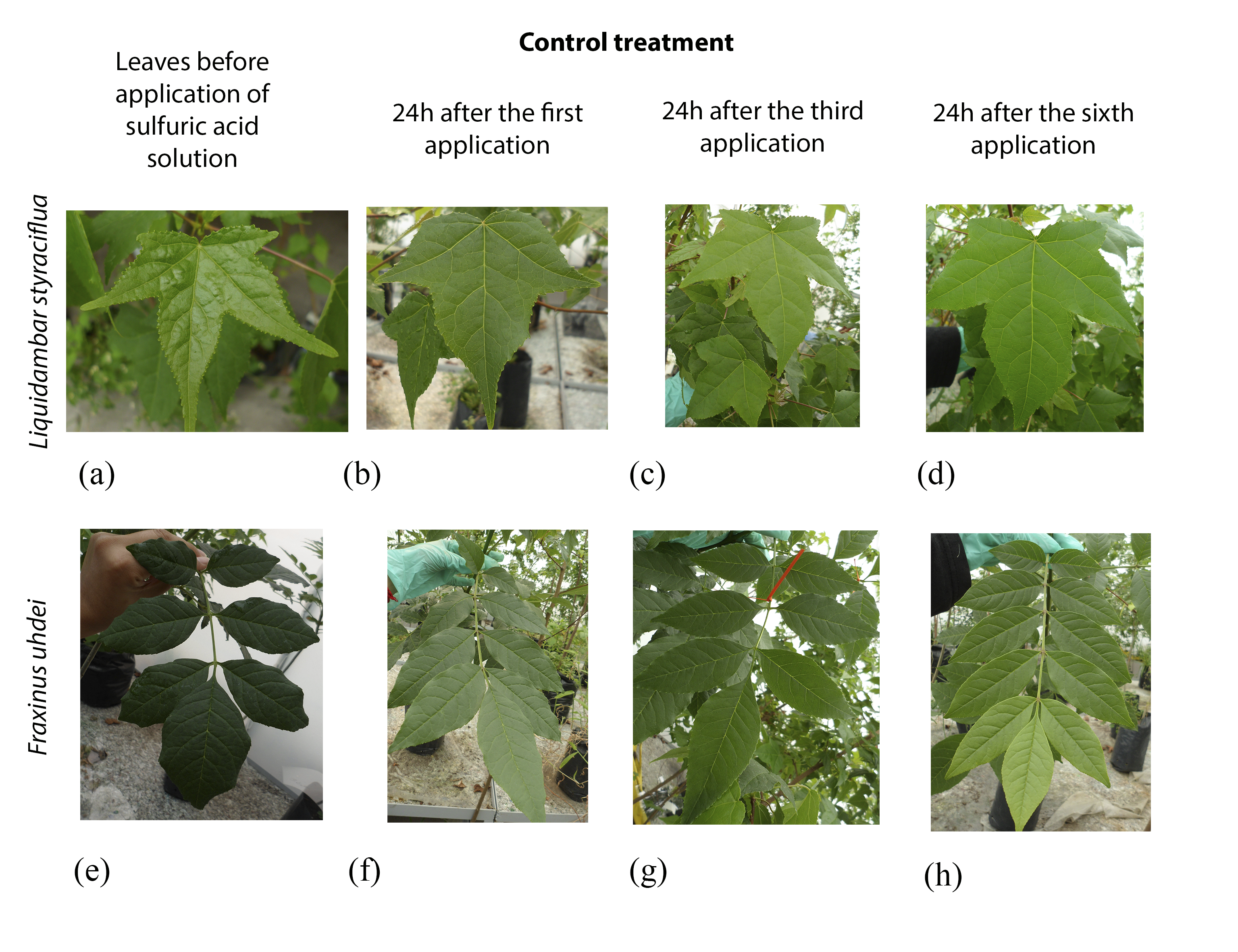

Supplement: Supplementary file 1 [file plants-09-00862-s001.zip › supplementary files/Figure S1_control treatment.tif]

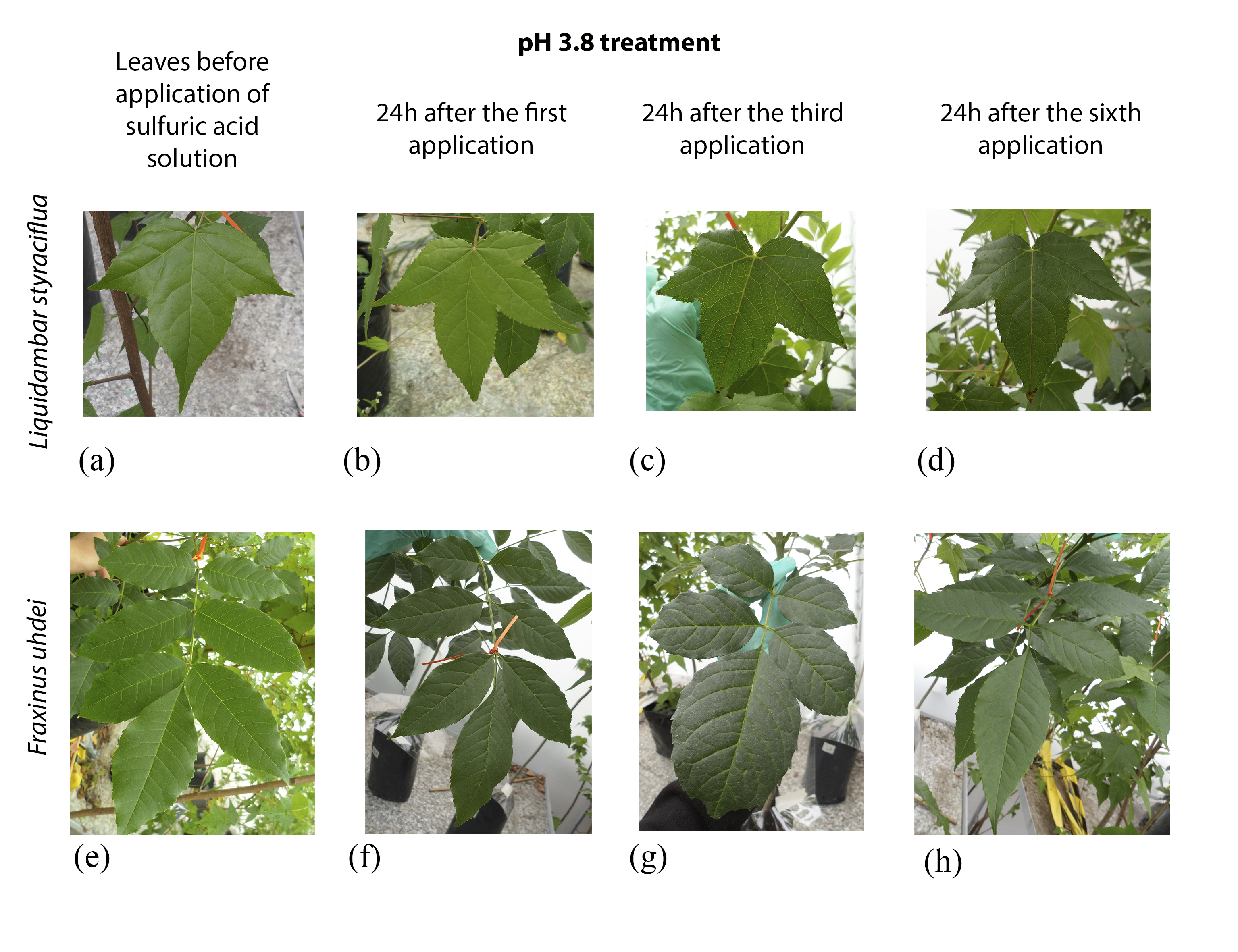

Supplement: Supplementary file 1 [file plants-09-00862-s001.zip › supplementary files/Figure S2_pH 3.8 treatment.tif]

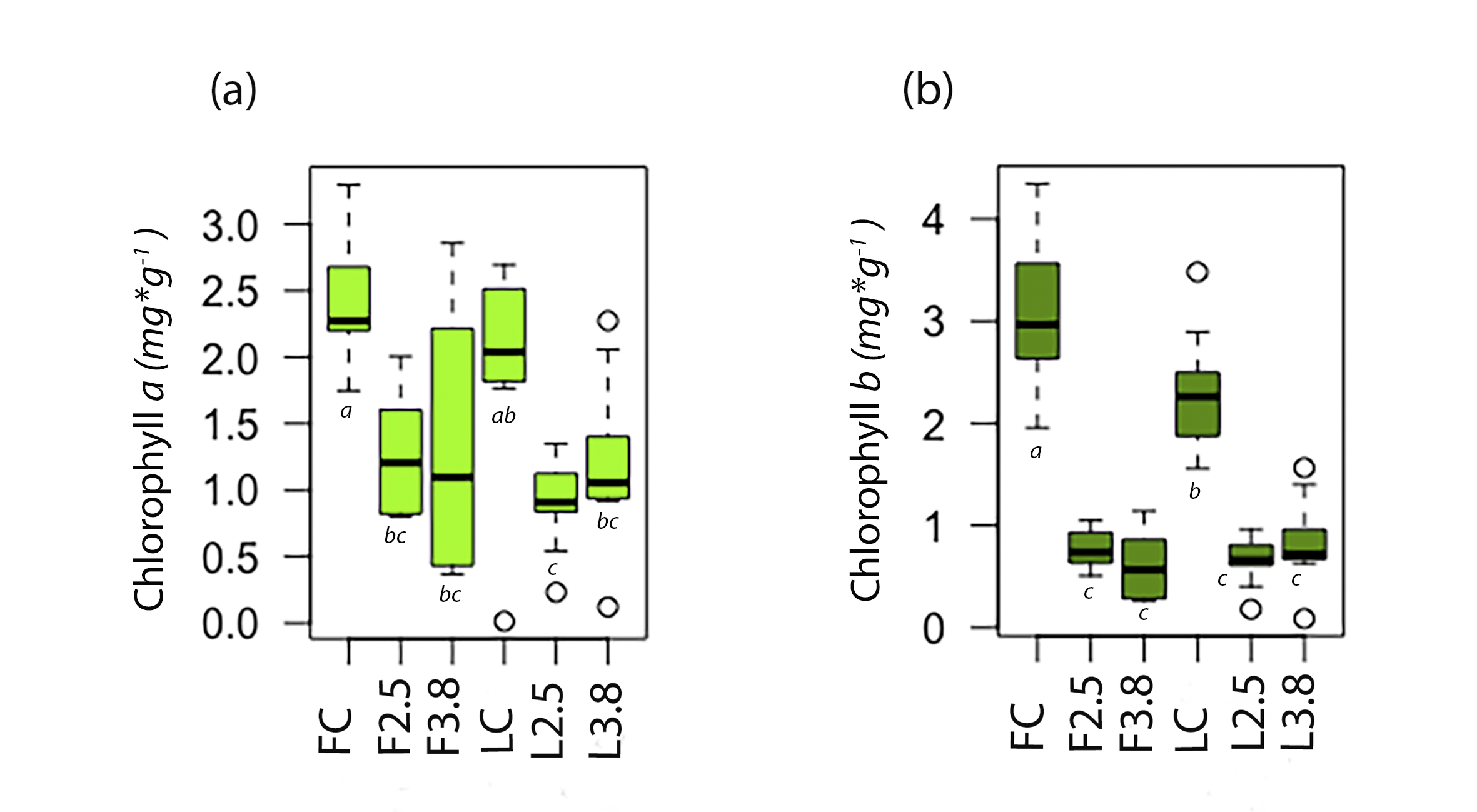

Supplement: Supplementary file 1 [file plants-09-00862-s001.zip › supplementary files/Figure S4_Chlorophyll content_fresh weight.tif]
